# A Longitudinal Observational Study on Lactation-Associated Changes in Procalcitonin, Protein Carbonyl Content, Asymmetric Dimethylarginine, and Symmetric Dimethylarginine in Dairy Cattle

**DOI:** 10.3390/vetsci12090895

**Published:** 2025-09-15

**Authors:** Giulia Sala, Matteo Castelli, Chiara Orsetti, Giovanni Armenia, Lucia De Marchi, Valentina Meucci, Micaela Sgorbini, Francesca Bonelli

**Affiliations:** 1 Department of Veterinary Sciences, University of Pisa, Via Livornese s.n.c., San Piero a Grado, 56122 Pisa, Italy; 2 Centro di Ricerche Agro-Ambientali “E. Avanzi”, University of Pisa, San Piero a Grado (PI), 56122 Pisa, Italy

**Keywords:** procalcitonin, protein carbonyl content, asymmetric dimethylarginine, symmetric dimethylarginine, dairy cattle

## Abstract

Procalcitonin (PCT), protein carbonyl content (PCC), asymmetric dimethylarginine (ADMA), and symmetric dimethylarginine (SDMA) have been proposed as promising biomarkers for detecting diseases in cattle. Their concentrations could potentially be influenced by lactation due to oxidative stress commonly observed during this period. This study aimed to evaluate plasma levels of PCT, PCC, ADMA, and SDMA at different stages of lactation in 21 healthy dairy cows: at 15 (T0), 60 (T1), and 150 (T2) days in milk (DIM). Clinically healthy Italian Holstein-Friesian cows were included, selected based on healthy dry periods and weekly veterinary checks during lactation. Blood samples were collected at each time point and biomarkers were measured using validated analytical methods. Data were analyzed using Friedman’s test and the *p* value was set at 0.05. Median (IQR) PCT values were 64.29 (40.00–143.23), 75.36 (40.00–161.47), and 77.50 pg/mL (40.00–120.18) at T0, T1, and T2, respectively. PCC medians were 0.17 (0.10–0.27), 0.14 (0.08–0.23), and 0.20 (0.08–0.22) nmol/mL/mg; ADMA values were 0.11 (0.09–0.15), 0.11 (0.09–0.13), and 0.10 µmol/L (0.09–0.14); and SDMA values were 0.11 (0.09–0.14), 0.12 (0.09–0.15), and 0.10 µmol/L (0.09–0.16). No statistically significant differences were observed between time points for any biomarker. These findings suggest that, despite physiological oxidative stress during lactation, these biomarkers remain stable in healthy cows. Therefore, establishing distinct reference ranges based on lactation stage may not be necessary.

## Simple Summary

Lactation is a physiologically demanding process for dairy cows, especially during the early post-calving period. This phase, known as the transition period, is associated with increased metabolic and immune challenges that can lead to oxidative stress and higher susceptibility to disease. In recent years, biomarkers have been studied for their ability to reflect the health and immune status of cows. Among these are procalcitonin, protein carbonyl content, and asymmetric and symmetric dimethylarginines. These biomarkers may help detect inflammation or stress in dairy cows. In this study, we investigated whether the levels of these four biomarkers change during different stages of lactation in healthy cows. Blood samples were taken from cows at three time points after calving. The results showed that the concentrations of these biomarkers remained stable and did not vary with the stage of lactation. This suggests that, in healthy animals, the stress of lactation does not significantly affect these markers. These findings contribute to our understanding of normal values in healthy cows and may support future research aimed at early detection of disease in dairy cattle.

## 1. Introduction

During lactation, dairy cows undergo physiological metabolic changes related to the lactation curve [1]. As a result, these changes can lead to an increase in oxidative stress and consequently impact the animal’s immunity [1]. During the productive cycle, the most critical phase for dairy cows is the transition period, defined as the phase spanning from 3 weeks before to 3 weeks after parturition [2]. During this period, nutrient requirements rise markedly to support fetal growth and the onset of colostrum and milk production, while dry matter intake simultaneously declines [3]. This imbalance therefore predisposes cows to significant metabolic, nutritional, physiological, and immunological changes [4]. Consequently, due to metabolic and oxidative stress combined with reduced immune defenses, cows are more susceptible to a variety of metabolic and infectious diseases in early lactation, which are directly related to the extent of metabolic alteration and the degree of immune dysfunction [5,6].

Biomarkers are valuable tools for assessing health status, supporting diagnosis and prognosis, monitoring treatment, and detecting organ toxicity or failure [7]. Moreover, in bovine medicine, biomarkers are studied for monitoring factors related to welfare and productivity [1]. Procalcitonin (PCT) and protein carbonyl content (PCC) are among the novel biomarkers extensively investigated in bovine species, especially in relation to various diseases. Elevated PCT levels in cattle have been associated with sepsis [8,9,10], bovine respiratory disease [11,12], clinical and subclinical mastitis [12,13,14,15], and other diseases requiring hospitalization [16]. Taken together, these findings highlight the potential of PCT as a biomarker for detecting and monitoring infections and inflammatory conditions in dairy cows. PCC is a biomarker for oxidative stress, which is common in dairy cows, especially during the periparturient period [17]. PCC as a biomarker has been studied in bovine mastitis [13,18], endometritis [19], and even in calves affected by *Theileria annulata* [20].

Nitric oxide (NO) is a pro-inflammatory mediator synthesized in endothelial cells, inducing oxidative stress and inflammation [21]. Dimethylarginines, including asymmetric dimethylarginine (ADMA) and symmetric dimethylarginine (SDMA), are derivatives of L-arginine that inhibit NO synthase activity [22,23]. ADMA, as an NOS inhibitor, affects NO production and vascular tone, with elevated levels associated with various diseases in both human and veterinary medicine [24,25,26,27,28]. In cattle, for instance, this marker was studied in premature calves affected by respiratory distress syndrome [29], in yaks adapted to high altitude [30], and in cows with mastitis [31]. SDMA, excreted through the kidneys, reflects changes in the glomerular filtration rate [24,32,33]. While widely utilized in veterinary medicine to stage chronic kidney disease (CKD) in dogs and cats [34,35], its plasma levels in bovines remain unstudied. Thus, ADMA and SDMA may serve as indirect biomarkers of oxidative stress and inflammation in bovines, potentially reflecting systemic inflammatory responses.

Despite these promising research efforts, the effect of changes in oxidative stress and immune status encountered by dairy cows during lactation on PCT, PCC, ADMA, and SDMA have not yet been investigated.

This study aimed to evaluate potential variations in the concentrations of PCT, PCC, ADMA, and SDMA in plasma samples from healthy cows at different stages of lactation. The working hypothesis was that oxidative stress resulting from the physiological changes associated with lactation could lead to an increase in the concentrations of these biomarkers.

## 2. Materials and Methods

This observational study was conducted at the dairy farm of the University of Pisa (Centro di Ricerche Agro-Ambientali “E. Avanzi”—CiRAA) between May 2023 and May 2024. Veterinary assistance was provided by the Large Animals Service of the Department of Veterinary Sciences (DSV) at the University of Pisa. The study was approved by the Institutional Animal Care and Use Committee of the University of Pisa (Prot. n: 18/2023 of 19 April 2023).

- Animals and Management

Lactating Italian Holstein Friesian cows from CiRAA underwent the same management practices. Lactating cows were housed in a free-stall barn with straw bedding, which was refreshed twice weekly and supplemented with clean straw every day. Each cow received the same total mixed ration twice a day and had *ad libitum* access to fresh water. Milking occurred twice a day in an 8-stall Herringbone milking parlor, with intervals of about 11 h between sessions (at 5 a.m. and 4 p.m.). Selective dry cow therapy was performed.

Additionally, udder health was monitored weekly by the veterinarian service, which included evaluation of teat end condition.

- Inclusion criteria

All cows calving during the study period at CiRAA were enrolled. To be included, cows had to meet the following criteria: (a) absence of clinical abnormalities, including physiological fertility evaluation in relation to postpartum phase; (b) udder health evaluation showing a California Mastitis Test (CMT) score < 1 [36], a Somatic Cell Count (SCC) < 200,000 cells/mL, and no alterations in the udder or milk [37]; and (c) negative results at bacteriological milk culture. Only Italian Holstein Friesian cows were included in the study.

Cows were visited weekly by veterinarians from the Large Animal Service of the DSV to perform underwent (a) clinical examination (GS) [38], including fertility evaluation (GA) [39,40]; (b) udder health evaluation (MC), including California Mastitis Test (CMT), Somatic Cell Count (SCC), and bacteriological analysis of milk at single quarter level. Moreover, any cow showing a health alert according to the Afifarm system was specifically examined.

Cows that developed any disease during lactation were excluded from the study.

- Sampling

To evaluate PCT, PCC, ADMA, and SDMA concentrations during lactation, blood samples were collected at 15 (T0), 60 (T1), and 150 (T2) DIM from the coccygeal vein in 3 lithium heparin tubes and centrifugated at 3000× *g* for 10 min. Obtained plasma was divided into 4 aliquots and stored at −80 °C until analysis. All samples were analyzed within 6 months from collection. To preserve the bioactivity of the samples, they were defrosted on ice for approximately 2 h before the analysis.

- Determination of plasma PCT, PCC, ADMA, and SDMA

Determination of biomarker content was performed at the Veterinary Pharmacology and Toxicology Laboratory, Department of Veterinary Sciences of Pisa.

PCT analysis: The plasmatic PCT levels were measured using a commercial ELISA kit specifically for bovine plasma (Bovine Procalcitonin ELISA Kit, Cusabio, Houston, TX, USA), with optical density measured at 450 nm. This kit has also been successfully applied to other bovine samples, further supporting the reliability of the assay [41]. The manufacturer reported a detection limit of 40 pg/mL. Results below the detection limit were confirmed, reported, and included in the statistical analysis as lod/2.

PCC Analysis: Plasmatic PCC was quantified following the method of Levine et al. [42]. Briefly, 1–10 mg/mL of a protein solution was mixed with 500 µL of 10 mM DNPH in 2 M HCl and incubated at room temperature for 1 h. After adding 500 µL of 10% TCA, samples were centrifuged for 3 min at 11,000 g. The resulting pellets were washed and centrifuged three times with ethanol–ethyl acetate (1:1). The precipitated protein was redissolved in 0.6 mL of guanidine solution, and insoluble materials were removed by centrifugation. Hydrazones were quantified via spectrophotometry at 340 nm absorbance (Synergy™ HTX, BioTek Instruments, Winooski, VT, USA). PCC was calculated using a molar absorption coefficient of 22,000 M^−1^ cm^−1^ and expressed as nmol/mL/mg of total protein. Total protein content was determined using the spectrophotometric method of Lowry et al. [43]) with bovine serum albumin (BSA) as the standard.

ADMA and SDMA Analyses: To determine the concentrations of these markers, high-performance liquid chromatography with fluorescence detection was used, as described by Teerlink [44]. Stock solutions of 1 mM arginine, homoarginine, ADMA, and SDMA in 10 mM HCl were mixed to create a working standard solution of 100 µM arginine, 10 µM homoarginine, ADMA, and SDMA, which was stored at −20 °C. After defrosting, a 40 µM working solution was prepared with PBS (10 mM sodium phosphate, 140 mM NaCl, pH 7.0). The derivatizing agent was prepared by mixing 10 mg orthophthalaldehyde (OPA) in 0.2 mL methanol, then adding 1.8 mL of 200 mM potassium borate buffer (pH 9.5) and 10 µL of 3-mercaptopropionic acid. The stock solution was diluted five times with borate buffer to create a working solution with final concentrations of 7.5 mM OPA and 11.5 mM 3-mercaptopropionic acid. Samples and standards were extracted in solid phase using a vacuum system. Each sample (0.2 mL) or standard was mixed with 0.1 mL of internal standard and 0.7 mL of PBS. Columns were washed with 1 mL of 100 mM HCl and 1 mL of methanol. Elution was performed in 3 mL tubes with 1 mL of a solvent mixture of concentrated ammonia/water/methanol (10/40/50). The solvent was evaporated with nitrogen, and 0.1 mL of OPA reagent was added. Samples were mixed and transferred to tubes for the automatic sampler and then moved to the Alliance separation module at 7 °C. The chromatography system included an Alliance 2690 XE separation module and a Model 474 fluorescence detector (Waters). Chromatography was performed on a Symmetry C18 column (3.9 × 150 mm; 5 µm granules; 100 Å porosity) with a guard column wrapped by the same stationary phase. Mobile phase A consisted of 50 mM potassium phosphate buffer (pH 6.5) containing 8.7% acetonitrile, while mobile phase B was a 50/50 mixture of acetonitrile and water. Separation occurred under isocratic conditions with 100% phase A at a flow rate of 1.1 mL/min and a column temperature of 30 °C. Remaining compounds were washed and eluted with a solvent after the last analyte was eluted. Fluorescence was measured at excitation and emission wavelengths of 340 and 455 nm, respectively. Detector sensitivity increased tenfold after arginine elution. Peak evaluation was based on the area under the curve. Intra- and inter-assay variation coefficients for ADMA and SDMA were below 11% and 15%, respectively. The limits of quantification, with a signal-to-noise ratio of 10, were 1 µg/dL for ADMA and 0.5 µg/dL for SDMA, using 0.2 mL of sample.

- Statistical analysis

The sample size was determined using G-power software (Ver. 3.1, Heinrich Heine University, Düsseldorf, Germany) and calculated with a non-parametric ANOVA test analysis. For the calculation, an effect size of 0.3 (low), a type I error (α) of 5%, a confidence interval of 9%, and a test power of 80% were utilized. The minimum number of cows required was 20.

Statistical analysis was performed with IBM SPSS Statistics v. 29.0 (IBM Corp., Armonk, NY, USA). Data distribution was investigated with the Shapiro–Wilk test, and we found that the data were not normally distributed. Therefore, the descriptive statistics of quantitative variables were reported as the median, 25th percentile, and 75th percentile, while qualitative variables were reported as frequencies and percentages.

The potential differences in PCT, PCC, ADMA, and SDMA medians during lactation in cows were evaluated with the Friedman test. Post hoc analysis was performed with Bonferroni adjustment. In addition, the variation in BCS across sampling times was investigated as a potential effect on biomarker concentrations using the Friedman test with Bonferroni adjustment. Statistical significance was considered for a *p*-value < 0.05.

## 3. Results

Forty-nine animals were eligible for the study; however, twenty-eight cows were excluded due to the development of diseases. Specifically, 10 cows (20.4%) developed mastitis, 10 cows (20.4%) had metritis, 6 cows (12.2%) developed a respiratory disease, and 2 cows (4.1%) had a foot disease. Thus, the study population was composed of 21 cows, sampled at three time points, for a total of 63 plasma samples analyzed.

**Figure 1:**
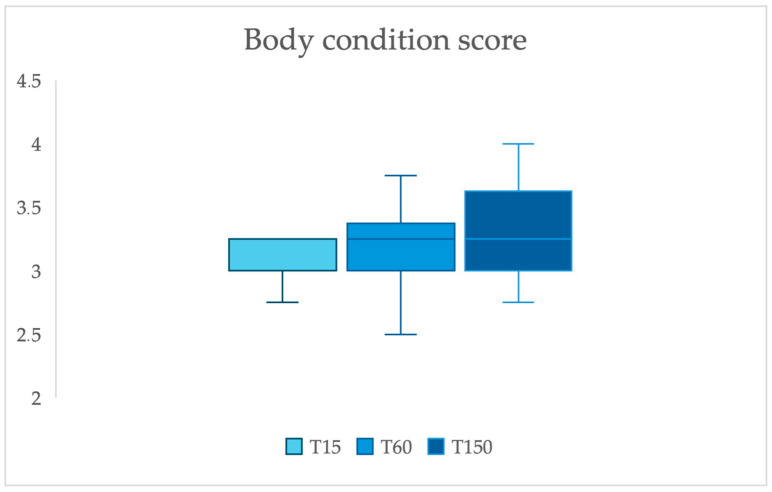
Box plot of Body Condition Score (BCS) of dairy cows included in the study at different time points during lactation (T15: 15 days in milk; T60: 60 days in milk; T150: 150 days in milk). No significant differences in BCS were observed across the sampling times (*p* value 0.343).

All cows included were Italian Holstein Friesian. Primiparous cows constituted 47.6% (10/21) of the sample, secundiparous cows accounted for 42.9% (9/21), and multiparous cows constituted 9.6% (2/21). Variation in BCS during lactation is reported in Figure 1, and no significant differences were observed across sampling times (*p* value 0.343).

The median values of biomarkers were as follows: PCT = 75.36 pg/mL (IQR: 40.00–129.64 pg/mL); PCC = 0.17 nmol/mL/mg (IQR 0.08–0.23 nmol/mL/mg); ADMA 0.105 µmol/L (IQR 0.094–0.144 µmol/L); and SDMA 0.110 µmol/L (IQR 0.090–0.149 µmol/L). The median values of biomarkers divided for sampling times are reported in Table 1 with the *p* value of the Friedman test. No differences during the sampling times were observed for all biomarkers analyzed in this study.

## 4. Discussion

The aim of the present study was to evaluate whether the stage of lactation in healthy cows could influence four biomarkers of interest: PCT, PCC, ADMA, and SDMA in the bovine species.

The PCT concentrations obtained in our study (64.29–77.50 pg/mL) are consistent with most studies using the same analytical method for PCT determination in healthy animals, including both adults (75.36 pg/mL) and calves (67.63–75.94 pg/mL) [13,45]. Only one study using the same methodology reported lower concentrations in adult animals (56.16 pg/mL) [15]. In contrast, studies employing different analytical techniques have reported markedly divergent results in adult animals (1166 pg/mL [14]; 200.1 pg/mL [16]). The comparison with the literature suggests that PCT levels in cattle may be influenced by the analytical method used.

Regarding the effect of lactation stage on PCT concentrations, no statistically significant differences were observed in the present study. To date, no previous studies have investigated this potential effect on PCT. A possible explanation for the lack of association between lactation and PCT levels is the specificity of PCT to increase in response to infectious diseases [46]. In bovine, PCT has shown good to excellent performance in the context of bacterial infections [13,15,16]. During both clinical and subclinical mastitis, it has proven to be a promising biomarker, with sensitivity ranging from 68.8% to 100% and specificity between 63.1% and 100% [13,14,15]. The animals included in this study were free of infectious diseases and remained healthy throughout lactation. This suggests that in animals that do not develop disease, PCT is not affected by oxidative stress or the pro-inflammatory state typically associated with the transition period [47]. In human medicine, it has been demonstrated that bacterial infections cause a significant increase in pro-inflammatory cytokines such as TNF-α and IL-1β, which subsequently induce the overexpression of the CALC-1 gene in various tissues, leading to PCT overproduction [46,48]. However, in a recent study investigating the role of oxidative stress during the transition period in dairy cows (−3 to +3 weeks from calving), only mild and non-significant changes in cytokines such as TNF-α and IL-6 were observed [47]. This supports the hypothesis that the slight and transient increase in inflammatory mediators during the transition period is not sufficient to trigger CALC-1 overexpression and, consequently, does not lead to a detectable rise in PCT levels in healthy animals.

PCCs in our study did not show statistically significant differences across the different stages of lactation, with a median value of 0.17 nmol/mL/mg. Previous studies on the PCC in dairy cows have reported concentrations ranging from 0.102 to 0.7 nmol/mL/mg in healthy animals [13,19], highlighting how this biomarker also yields variable results across studies. Regarding the effect of lactation, one previous study investigated the influence of DIM on PCCs and similarly found no significant differences [13].

This result was unexpected. In fact, Kuhn et al. [49] demonstrated that oxidative status changes throughout lactation, with increased oxidative activity in early lactation that decreases in mid and late lactation. PCC concentrations would be expected to rise in response to oxidative stress as PCC reflects oxidative modifications to proteins induced by reactive oxygen species (ROS), making it a useful and relatively stable marker of oxidative damage [50,51]. ROS, including superoxide, hydroxyl radicals, and hydrogen peroxide, are highly reactive molecules derived from oxygen metabolism. When ROS production exceeds antioxidant capacity, proteins become targets for oxidative modification, especially through the formation of carbonyl groups on amino acid side chains. As these modifications accumulate, they can be quantified to assess the extent of oxidative stress [50,51].

A possible explanation for the lack of association between lactation stage and PCCs may be that, although oxidative stress is physiologically present, it is counterbalanced by antioxidant defense mechanisms, preventing the development of measurable oxidative damage. This supports the observation that the animals remained clinically healthy throughout lactation.

No variation in relation to different lactation stages was observed for either ADMA or SDMA, with median values of 0.105 µmol/L and 0.110 µmol/L, respectively. These values are consistent with previous studies, where ADMA ranged from 0.11 to 0.53 µmol/L and SDMA ranged from 0.11 to 0.73 µmol/L in healthy lactating cows [31,52]. Both ADMA and SDMA have been scarcely investigated in cattle, and the available data are often inconsistent [31,52,53,54,55].

From a physiological perspective, the metabolic and inflammatory challenges of early lactation could be expected to influence circulating ADMA and SDMA concentrations given their involvement in nitric oxide metabolism, oxidative stress, and immune responses. However, in our study, such changes were not observed. A possible explanation lies in the health status of the animals. All cows included remained clinically healthy throughout lactation, and no overt inflammatory or infectious conditions were detected. In the absence of a strong systemic inflammatory stimulus, the physiological increase in oxidative stress typical of early lactation may not have been sufficient to impair DDAH activity (the enzyme responsible for ADMA degradation) or significantly alter arginine metabolism and transport [56]. This could explain the stability of ADMA and SDMA concentrations across lactation stages.

This study presents some limitations. First, although the literature has shown that lactation, particularly the transition period, is associated with increased oxidative stress in dairy cows, the absence of direct oxidative stress biomarkers or inflammatory cytokines in our study prevented us from confirming whether such stress occurred in the included animals and from corroborating the potential mechanisms underlying biomarker stability. Based on existing studies, we speculate that animals likely experienced a certain degree of oxidative stress; however, this assumption could not be directly verified. Future studies should therefore integrate direct oxidative stress markers and cytokines together with PCT, PCC, ADMA, and SDMA in order to better clarify their interrelationships and validate their role in monitoring physiological and pathological conditions in dairy cows. Secondly, the strict inclusion of only clinically healthy cows throughout the entire lactation period limited the possibility of evaluating these biomarkers as early indicators of disease. This choice was intentional in order to avoid the potential confounding effect of pathological conditions on biomarker concentrations. Nevertheless, this limitation was beyond the scope of the present study. Another limitation of the present study is that parity was not included as a fixed effect in the statistical model. Although its distribution within the cohort was reported, the potential influence of parity on biomarker concentrations cannot be excluded. In addition, while BCS was evaluated across sampling times and no significant differences were observed, its potential role as a covariate was not further investigated. Both factors should be further explored in future studies. Moreover, the sampling schedule was not tightly defined during the early stages of lactation, which may have limited the ability to capture rapid physiological changes. Extending and refining the timing of sample collection, particularly in early lactation, could be the goals of future studies. Finally, although the sample size was determined a priori using a power analysis and met the calculated requirements, the relatively limited number of animals still represents a constraint. This may reduce the generalizability of the findings, and larger cohorts will be needed in future studies to validate these results.

## 5. Conclusions

This study found no significant differences in the concentrations of PCT, PCC, ADMA, or SDMA across different stages of lactation in healthy dairy cows. These findings suggest that, in the absence of clinical disease, the physiological oxidative stress associated with lactation was not sufficient to alter these biomarkers. The results provide a basis for future research aimed at defining normal reference ranges and exploring their potential role in early disease detection.

## Figures and Tables

**Table 1:** Descriptive statistics (median and 25° and 75° percentiles) of procalcitonin (PCT), protein carbonyl content (PCC), asymmetric dimethylarginine (ADMA), and symmetric dimethylarginine (SDMA), along with the *p*-value. DIM: days in milk.

	15 DIM	60 DIM	150 DIM	*p* Value
PCT (pg/mL)	64.29 (40.00–143.23)	75.36 (40.00–161.46)	77.50 (40.00–120.18)	0.225
PCC (nmol/mL/mg)	0.17 (0.10–0.27)	0.14 (0.08–0.22)	0.19 (0.08–0.22)	0.432
ADMA (µmol/L)	0.111 (0.085–0.155)	0.104 (0.091–0.134)	0.104 (0.095–0.141)	0.867
SDMA (µmol/L)	0.110 (0.086–0.137)	0.127 (0.091–0.157)	0.100 (0.087–0.164)	0.953

## Data Availability

The data presented in this study are available on request from the corresponding author due to privacy restrictions related to farm data.

## Abbreviations

The following abbreviations are used in this manuscript:

PCT	Procalcitonin
PCC	Protein carbonyl content
ADMA	Asymmetric dimethylarginine
SDMA	Symmetric dimethylarginine
BCS	Body condition score
SCC	Somatic cell count
CMT	California Mastitis Test
NO	Nitric oxide
NOS	Nitric oxide synthase
iNOS	Inducible nitric oxide synthase
DDAH	Dimethylarginine dimethylaminohydrolase
DIM	Days in milk
ROS	Reactive oxygen species
